# The cost of data collection for performance monitoring in hospitals: protocol for a systematic review

**DOI:** 10.1186/2046-4053-3-65

**Published:** 2014-06-16

**Authors:** Brenda Gannon, Cheryl Jones, Abel Wakai, Ronan O’Sullivan

**Affiliations:** 1 Centre for Health Economics, The University of Manchester, Oxford Road, M13 9PL Manchester, UK; 2 Department of Emergency Medicine, Beaumont Hospital, Dublin; Emergency Care Research Unit (ECRU), Beaumont Road, Beaumont, Dublin 9,Division of Population Health Sciences, Royal College of Surgeons in Ireland (RCSI), 123 St. Stephen’s Green, Dublin 2, Ireland; 3 Paediatric Emergency Research Unit (PERU), National Children’s Research Centre, Dublin, Crumlin, Dublin 12, Ireland; 4 Department of Emergency Medicine, Cork University Hospital, Wilton, Cork, Ireland

**Keywords:** Quality indicators, Healthcare, Quality improvement, Hospitals

## Abstract

**Background:**

Key performance indicators (KPIs) are used to identify where organisational performance is meeting desired standards and where performance requires improvement. Valid and reliable KPIs depend on the availability of high-quality data, specifically, the relevant minimum data set (MDS; the core data identified as the minimum required to measure performance for a KPI) elements. However, the feasibility of collecting the relevant MDS elements is always a limitation of performance monitoring using KPIs. Preferably, data should be integrated into service delivery, and where additional data are required that are not currently collected as part of routine service delivery, there should be an economic evaluation to determine the cost of data collection. The aim of this systematic review is to synthesise the evidence base concerning the costs of data collection in hospitals for performance monitoring using KPIs, and to identify hospital data collection systems that have proven to be cost minimising.

**Methods/Design:**

Electronic databases will be systematically searched for publications in English that examine the cost of data collection within a hospital context. The database searches will be supplemented by searching through citations and references. Screening of both titles and abstracts will be done by two independent reviewers. All disagreements will be resolved by an independent third reviewer. Data analysis will be completed and reported in a narrative review.

**Discussion:**

This review will cohere the evidence base regarding cost-minimising hospital data collections systems for performance monitoring and if these are associated with potential benefits for patients.

**Systematic review registration:**

PROSPERO CRD42014007450

## Background

Key performance indicators (KPIs) are used to identify where organisational performance is meeting desired standards and where performance requires improvement. KPIs enable the public, service users and healthcare providers alike to have reliable information on current and desired standards in healthcare services (HIQA, [1]). However, the feasibility of collecting the relevant minimum data set (MDS; the core data identified as the minimum required to measure performance for a KPI) elements is always a limitation of performance monitoring using KPIs. For example, in a pilot feasibility analysis of four potential emergency department (ED) KPIs, approximately half of the relevant MDS items were missing in the ED clinical records [2].

The reporting burden of capturing the relevant MDS elements should not outweigh the value of information when using KPIs for performance monitoring (HIQA, [1]). Preferably, data should be integrated into service delivery, and where additional data are required that are not currently part of service delivery, there should be an economic evaluation to determine the cost of collecting all the relevant MDS elements (HIQA, [1]). There is, therefore, a need for a systematic review which synthesises and coheres with the evidence base regarding economic analyses of hospital data collection for performance monitoring purposes.

## Methods/Design

### Research objectives

The aim of this review is to synthesise the evidence base concerning the costs of hospital data collection for performance monitoring using key performance indicators (KPIs), and to identify hospital data collection systems that have proven to be cost-minimising. There are two main objectives:

1. To identify published economic evaluations and cost studies regarding hospital data collection for performance monitoring purposes; and

2. To identify and summarise the methods used to evaluate hospital data collection for performance monitoring purposes.

### Research questions

The main questions of this review are as follows:

1. What literature exists on the economic analysis of hospital data collection for performance monitoring purposes using KPIs?

2. Once identified, how do these studies measure the economic costs and health benefits of hospital data collection for performance monitoring purposes?

### Systematic review

The research questions will be addressed using a systematic review of the qualitative and quantitative literature. The conclusions will then be summarised in a narrative review using the evidence found. The components of this systematic review are:

#### Inclusion criteria

All economic analysis and costing studies regarding hospital data collection for performance monitoring purposes will be included in the review. See Additional file 1: Table S1 for the inclusion criteria form.

(i) Types of studies

The types of studies that are to be identified are economic evaluations and cost or feasibility studies regarding hospital data collection for performance monitoring purposes using KPIs. For the purpose of this review, the definition of KPI will include any variable or a synonym of an indicator used to measure key areas of a service for performance monitoring purposes. Therefore, studies examining quality-of-care indicators and clinical indicators will be screened for inclusion.

(ii) Types of participant

There are broadly speaking two types of participant that are routinely involved in hospital data collection systems for performance monitoring: clinical (doctors, nurses, and allied health professionals, such as physiotherapists, respiratory therapists and occupational therapists) and non-clinical staff (for example, administrators, clerical staff, managers and information technology staff).

(iii) Types of interventions

Hospitals may have varied ways of recording and collecting data for performance monitoring purposes using KPIs. All types of interventions (as defined by the study authors) that are used to record and collect data will be included in this review.

#### Exclusion criteria

Studies that will be excluded from the review will be those cost and economic analyses and feasibility studies that do not focus on data collection in the hospital setting.

#### Searching

To identify relevant research, a search strategy has been produced and can be found in Additional file 2: Table S2. The search strategy will then be applied to appropriate databases to search for potentially eligible studies. Databases that will be searched include Medline, Embase and CINAHL via the Ovid SP website. The titles and abstracts of all identified studies potentially eligible for inclusion in the review will be searched. Full-text versions of these potentially eligible studies will be obtained. Additional efforts to identify more potentially eligible studies for inclusion will be made from the following data sources:

1. Reference lists from included studies; and

2. Published reports by the Departments of Health based in the United Kingdom, Ireland, the United States, Canada and Australia.

#### Methods for study selection

The screening process for study selection will be completed in two stages. Stage one will include screening the titles and abstracts of each publication against the inclusion criteria. The studies that will be selected for the review will meet all of the inclusion criteria requirements. Stage two will involve screening the full-text version of the publication to confirm whether or not the study should be included in the final review. Stages one and two will be conducted by two investigators (BG and CJ) independently, and a comparison of included and excluded studies will be carried out. If there are discrepancies with any inclusion or exclusion decisions, and the two investigators who independently screen potentially eligible studies cannot reach a consensus, the disagreements will be resolved through discussion and consultation with a third reviewer (AW).

#### Primary abstract screening

An inclusion/exclusion form (see Additional file 1: Table S1) will be used to initially determine whether or not a study meets the requirements to satisfy inclusion based on reading the Abstract and Conclusion sections. Initial inclusion criteria will consist of:

1. Economic evaluations that address the cost of hospital data collection for performance monitoring using KPIs; and

2. Studies published in English.

If it is not clear whether a study will be rejected by the reviewers from the initial screening of the title and abstract, the full text will be obtained and further evaluation of the study will be carried out.

#### Methods for data extraction

One author (CJ) will extract data using a tailored data collection form that includes information concerning the name of the first author, year of publication, study design, study population and study setting. A second investigator (BG) will then verify the extracted data. Data that will be extracted from studies will include cost data, potential health benefits, data collection systems that have been/are used and if any of these systems have been found to be cost minimising. See Additional file 3: Table S3 for the full list of information that will be extracted.

#### Quality assessment

Each study identified will be assessed for its quality using the Consolidated Health Economic Evaluation Reporting Standards (CHEERS) guidelines published by the International Society for Pharmacoeconomics and Outcomes Research (ISPOR) (CHEERS, [3]) (see Additional file 4: Table S4 for full list). One researcher (CJ) will conduct the quality assessment and a second researcher (BG) will review and highlight any discrepancies. If there are discrepancies concerning quality assessment decisions and the two investigators performing the assessment cannot reach a consensus, the disagreements will be resolved through discussion and consultation with a third reviewer (AW).

#### Data synthesis

Data will not be synthesised using quantitative techniques. The extracted data will be summarised in tables and a narrative review will be prepared. This will include an assessment of what systems hospitals currently use to collect data for the purpose of performance monitoring using KPIs, a description of the alternative (traditional) systems used to collect data and a description of the costs and benefits associated with hospital data collection.

#### Subgroup analysis

A subgroup analysis will be performed on hospital data collection for performance monitoring purposes in the emergency care setting versus the non-emergency care setting, because hospital-provided care can be broadly divided into these two categories.

## Discussion

The main aim of this review is to collate the evidence regarding cost-minimising hospital data collection systems for performance monitoring in emergency departments. The review will also identify the benefits associated with improved data collection systems, to both patients and providers of health care. The results of the review will contribute to the development of KPIs with the aim of improving the quality of care provided in Irish Hospitals. We anticipate that the review will be useful to a variety of stakeholders who have an interest in improving the quality of care in hospitals. This research aims to provide a comprehensive review of evidence on the extent to which data collection systems in hospitals is cost-effective.

## Abbreviations

ED: emergency department; KPIs: key performance indicators; MDS: minimum data set.

## Competing interests

The authors declare that they have no competing interests.

## Authors’ contributions

All authors read and approved the final manuscript.

## Source of funding

Health Research Board Ireland, Health Research Awards 2012(HRA_HRS/2012/18).

## Supplementary Material

Additional file 1: Table S1 Inclusion criteria. Click here for file

Additional file 2: Table S2 Search Terms. Click here for file

Additional file 3: Table S3 Data extraction sheet. Click here for file

Additional file 4: Table S4 Quality Assessment Checklist. Click here for file
