# Supplementary material for: The cost of data collection for performance monitoring in hospitals: protocol for a systematic review
Source: Syst Rev. 2014 Jun 16;3:65. doi: 10.1186/2046-4053-3-65 (PMC4065583; doi:10.1186/2046-4053-3-65)
Supplement: Additional file 1: Table S1 — Inclusion criteria. [file 2046-4053-3-65-S1.doc]

Additional file 1

**Table S1.** Inclusion criteria

| Inclusion Criteria | Outcome |  |
| --- | --- | --- |
|  | Yes | No |
| Economic Evaluation or Cost/ Feasibility study |  |  |
| Data Collection or Quality/Clinical Indicator study |  |  |
| Hospital/Secondary care context |  |  |
| English or English translation |  |  |
| Assessment | In | Out |
| Tick |  |  |

Further instructions for inclusion:

For the purpose of this review, the definition of KPI will include any variable or a synonym of an indicator used to measure key areas of a service for performance monitoring purposes. Therefore, studies examining quality-of-care indicators and clinical indicators will be screened for inclusion.
