# Supplementary material for: The cost of data collection for performance monitoring in hospitals: protocol for a systematic review
Source: Syst Rev. 2014 Jun 16;3:65. doi: 10.1186/2046-4053-3-65 (PMC4065583; doi:10.1186/2046-4053-3-65)
Supplement: Additional file 2: Table S2 — Search Terms. [file 2046-4053-3-65-S2.doc]

Additional file 2

**Table S2. Search Terms**

1. Physicians

2. Nurses

3. Medical Staff, hospital

4. Exp Hospital

5. Exp Hospital Department

6. Or / 1 - 5

7. Hospital, animal

8. 6 not 7

9. Exp Data Collection

10. Exp Medical Records Systems, Computerised

11. Exp Quality Indicators, Healthcare

12. Public Health Administration

13. Or/ 9 - 12

14. 8 and 13

15. Economics/

16. exp “costs and cost analysis”/

17. Economics, Dental/

18. exp economics, hospital/

19. Economics, Medical/

20. Economics, Nursing/

21. Economics, Pharmaceutical/

22. (economic$ or cost or costs or costly or costing or price or prices or pricing or pharmacoeconomic$).ti,ab.

23. (expenditure$ not energy).ti,ab.

24. value for money.ti,ab.

25. budget$.ti,ab.

26. or/15-25

27. ((energy or oxygen) adj cost).ti,ab.

28. (metabolic adj cost).ti,ab.

29. ((energy or oxygen) adj expenditure).ti,ab.

30. or/27-29

31. 26 not 30

32. letter.pt.

33. editorial.pt.

34. historical article.pt.

35. or/32-34

36. 31 not 35

37. exp animals/ not humans/

38. 36 not 37

39. bmj.jn.

40. “cochrane database of systematic reviews”.jn.

41. health technology assessment winchester england.jn.

42. journal of medical economics.jn.

43. or/39-42

44. 38 not 43
