# Supplementary material for: The cost of data collection for performance monitoring in hospitals: protocol for a systematic review
Source: Syst Rev. 2014 Jun 16;3:65. doi: 10.1186/2046-4053-3-65 (PMC4065583; doi:10.1186/2046-4053-3-65)
Supplement: Additional file 3: Table S3 — Data extraction sheet. [file 2046-4053-3-65-S3.doc]

Additional file 3

**Table S3.** Data extraction sheet

| Data to be Extracted | Notes to the Reviewer |
| --- | --- |
| Title of study |  |
| Author |  |
| Year of Publication |  |
| Study of Data Collection |  |
| Type of Study (CEA/CBA) |  |
| Setting |  |
| Time |  |
| Study objective clearly stated |  |
| Study Methodology used |  |
| Inclusion of Sufficient Data |  |
| Data Source |  |
| Other Relevant Details |  |
| Population/ Context |  |
| Clinical Staff |  |
| Non-clinical Staff |  |
| Type of Hospital Department |  |
| Data Collection |  |
| How Data is recorded? |  |
| How data is collected? |  |
| What technology systems are used? |  |
| Costs |  |
| How much? |  |
| Currency |  |
| Price Year |  |
| Health Benefits |  |
| What are the Health Benefits being reported? |  |
| Valuation of Health Benefits for example, QALYs |  |
| Results |  |
| Cost effective? |  |
| ICER? |  |
| Overall conclusion |  |
