# Supplementary material for: The cost of data collection for performance monitoring in hospitals: protocol for a systematic review
Source: Syst Rev. 2014 Jun 16;3:65. doi: 10.1186/2046-4053-3-65 (PMC4065583; doi:10.1186/2046-4053-3-65)
Supplement: Additional file 4: Table S4 — Quality Assessment Checklist. [file 2046-4053-3-65-S4.doc]

Additional file 4

**Table S4.** Quality Assessment Checklist

| Section/ Item | Item No. | Recommendation | Reported on page no./line no. |
| --- | --- | --- | --- |
| Title and Abstract |  |  |  |
| Title | 1 |  |  |
| Abstract | 2 |  |  |
| Introduction |  |  |  |
| Background and objectives | 3 |  |  |
| Methods |  |  |  |
| Target Population and subgroups | 4 |  |  |
| Setting and Location | 5 |  |  |
| Study Perspective | 6 |  |  |
| Comparators | 7 |  |  |
| Time Horizon | 8 |  |  |
| Discount Rate | 8 |  |  |
| Choice of Health Outcomes | 10 |  |  |
| Measurement of Effectiveness | 11a |  |  |
| Measurement and valuation of preference - based outcomes | 11b |  |  |
| Estimating recourse and costs | 12 |  |  |
| Currency, price date and conversion | 13a |  |  |
| Choice of Model | 13b |  |  |
| Assumptions | 16 |  |  |
| Analytic Methods | 17 |  |  |
| Results |  |  |  |
| Study Parameters | 18 |  |  |
| Incremental Costs and Outcomes | 19 |  |  |
| Characterising Uncertainty | 20 |  |  |
| Characterising Heterogeneity | 21 |  |  |
| Discussion |  |  |  |
| Study findings, limitations, generalisability and current knowledge | 22 |  |  |
| Other |  |  |  |
| Source of funding | 23 |  |  |
| Conflicts of interest | 24 |  |  |

Source: CHEERS checklist, ISPOR (2013)
